# Flexible Organic Solar Cells: Progress and Challenges

**DOI:** 10.1002/smsc.202100001

**Published:** 2021-05-04

**Authors:** Yanna Sun, Tao Liu, Yuanyuan Kan, Ke Gao, Bo Tang, Yuliang Li

**Affiliations:** ^1^ Science Center for Material Creation and Energy Conversion Institute of Frontier and Interdisciplinary Science Shandong University Qingdao 266237 P. R. China; ^2^ College of Chemistry Chemical Engineering and Materials Science Key Laboratory of Molecular and Nano Probes Ministry of Education Collaborative Innovation Center of Functionalized Probes for Chemical Imaging in Universities of Shandong Institute of Materials and Clean Energy Shandong Provincial Key Laboratory of Clean Production of Fine Chemicals Shandong Normal University Jinan 250014 P. R. China; ^3^ Department of Chemistry Guangdong-Hong Kong-Macao Joint Laboratory of Optoelectronic and Magnetic Functional Materials Energy Institute and Hong Kong Branch of Chinese National Engineering Research Center for Tissue Restoration & Reconstruction Hong Kong University of Science and Technology Clear Water Bay Kowloon Hong Kong 999077 P. R. China; ^4^ Beijing National Laboratory for Molecular Sciences (BNLMS) Institute of Chemistry Chinese Academy of Sciences Beijing 100190 P. R. China

**Keywords:** electronic devices, flexible organic photovoltaic devices, flexible transparent electrodes, performance of organic solar cells

## Abstract

Compared with inorganic photovoltaic technologies, flexibility is the most prominent feature of organic solar cells (OSCs). Flexible OSCs have been considered as one of the most promising directions in the OSC field, and have drawn tremendous attention in recent years. However, the power conversion efficiencies (PCEs) of flexible OSCs still lag behind those of their rigid counterparts. To further improve the performance of flexible OSCs, it is of great necessity for synergistic efforts to optimize flexible transparent electrodes (FTEs), photoactive materials, electrode buffer layers, and device structure engineering. Herein, the recent progress in flexible OSCs from the perspective of FTEs, including indium tin oxides, carbon nanomaterials, conducting polymers, silver nanowires, and ultrathin metal films and metal meshes, is summarized. In addition, the photoactive materials and electrode buffer layers in flexible OSCs are discussed to reveal the effects of material engineering and interface modification. Finally, a discussion of the future outlook and challenges of flexible OSCs is presented.

## Graphical abstract

The recent progress in flexible organic solar cells (OSCs) based on various flexible transparent electrodes (FTEs) is reviewed. Moreover, a detailed discussion of OSCs' performance with various FTEs and photoactive materials is provided to unravel the underlying structure‐property relationship. In the last section, the opportunities and challenges of flexible OSCs are discussed.

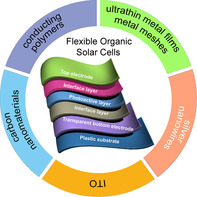

## 1 Introduction

Organic solar cells (OSCs) possess the unique merits of lightweight, intrinsic flexibility, large‐area printing fabrication, and low cost, which have been regarded as one of the most promising clean energy solutions.^[^ ^1^, ^2^, ^3^, ^4^, ^5^, ^6^, ^7^ ^]^ Recently, the rigid OSCs have realized impressive power conversion efficiencies (PCEs) of over 18% for single‐junction devices and 17% for tandem devices.^[^ ^8^, ^9^, ^10^, ^11^, ^12^, ^13^, ^14^, ^15^ ^]^ Compared with inorganic photovoltaic technologies, flexibility is considered the most prominent feature in OSCs.^[^ ^16^, ^17^, ^18^, ^19^, ^20^ ^]^ However, the performance of flexible OSCs still significantly lags behind that of the rigid devices, which only obtain relatively low PCEs of ≈16%. Therefore, much more attention should be paid to realizing highly effective flexible OSCs.

**Figure 1:**
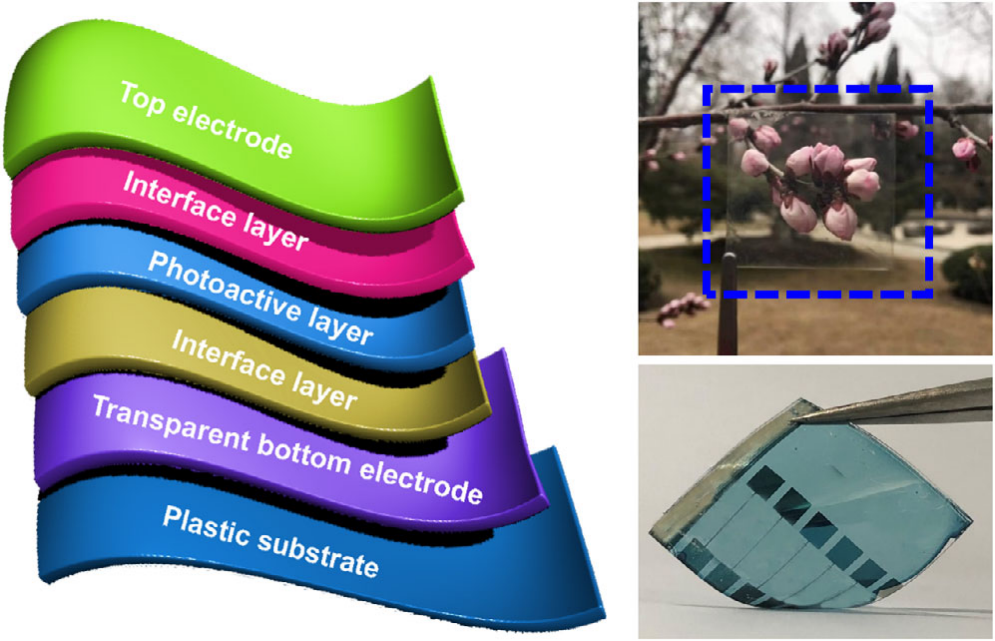
Left: the typical device structure of flexible OSCs. Right: photographs of a FTE and a flexible device.

A typical flexible OSC device is composed of a flexible transparent electrode (FTE), one photoactive layer, two electrode buffer layers, and a top electrode (**Figure** **1**). Note that FTE is the inevitable and fundamental component and plays a decisive role in device performance and mechanical flexibility. To achieve highly efficient flexible OSCs, high‐quality FTEs should offer the following characteristics simultaneously: 1) low sheet resistance to reduce device series resistance; 2) high transmittance to allow active layer to absorb more photons; 3) appropriate work function (WF) to guarantee efficient charge extraction; 4) low surface roughness to avoid leakage current or even electrical shortage; 5) excellent mechanical stability to maintain PCE during bending; and 6) low cost and solution processability to be compatible with roll‐to‐roll process for high throughput production.^[^ ^16^, ^19^, ^21^ ^]^ Indium tin oxides (ITO), as the most common transparent electrode (TE) material, could not fulfill the requirements of high‐quality FTEs due to its high cost and intrinsic brittleness.^[^ ^22^ ^]^ Thus, novel FTE materials suitable for efficient flexible OSCs, such as carbon nanomaterials,^[^ ^23^, ^24^ ^]^ conducting polymers,^[^ ^25^, ^26^ ^]^ metal nanowires,^[^ ^21^, ^27^ ^]^ ultrathin metal films and metal meshes,^[^ ^28^, ^29^ ^]^ and so on, have been deeply investigated, which will be further discussed in Sections 2, 3, 3.1, 3.1.1, 3.1.2, 3.2, 4, 5, 6.

**Figure 2:**
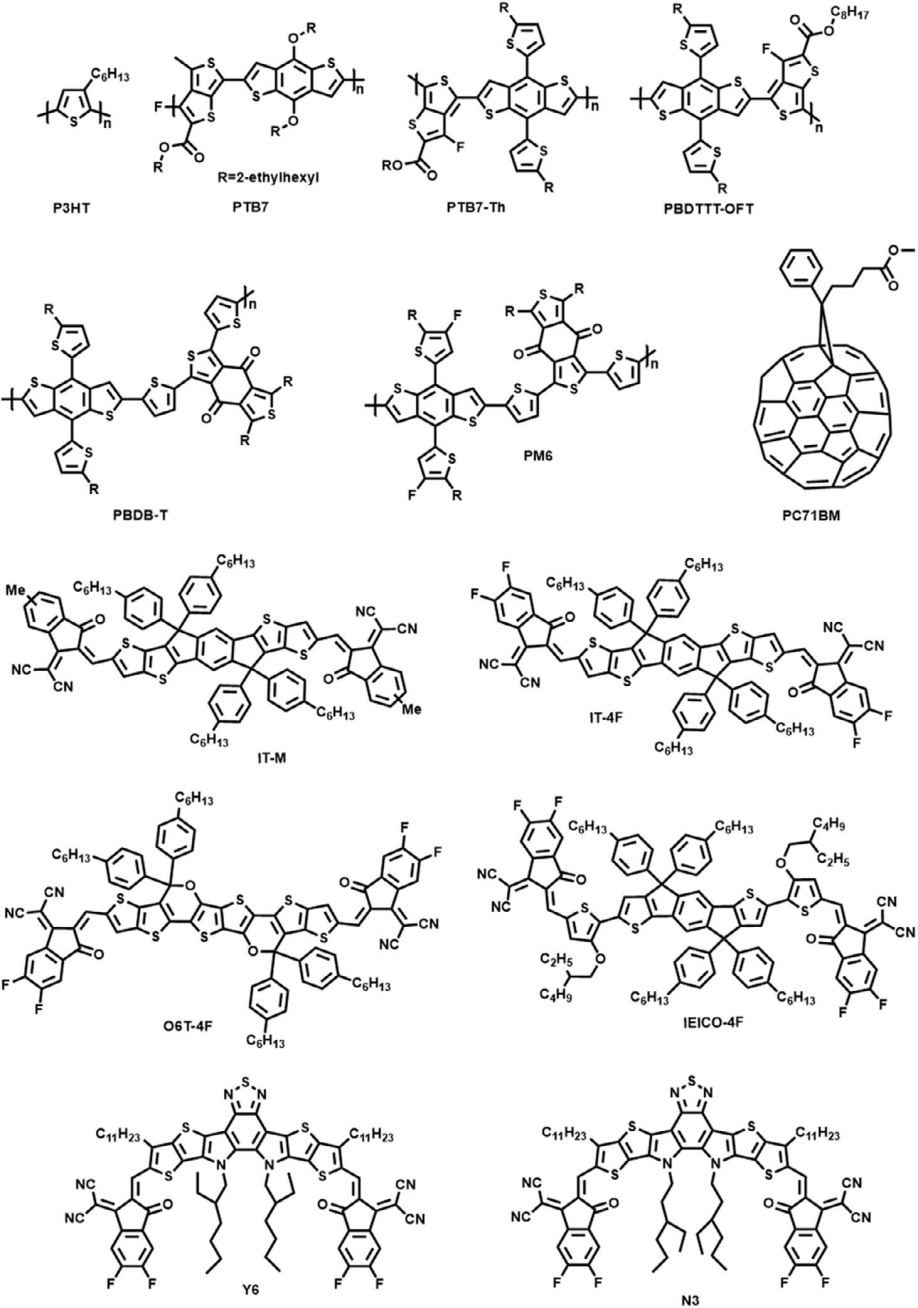
Chemical structures of some typical donor and acceptor materials used in flexible OSCs.

Electrode buffer layers and photoactive materials are the other two important factors in determining the photovoltaic performance of flexible OSCs. The electrode buffer layer (anode and cathode interface layer) between active layer and electrode ensures efficient charge transportation and collection. Given that the compatibility with the roll‐to‐roll technique and plastic substrates in flexible OSCs, developing thick‐film and low‐temperature processed interface materials is of great importance.^[^ ^21^, ^27^ ^]^ In addition, the active layer involves donor and acceptor materials, and is the photoactive part for converting solar light to electricity. In other words, the active layer materials are a prerequisite to realize high‐efficiency OSCs regardless of device types. With this in mind, intense efforts have been made to molecular design for efficient photoactive materials.^[^ ^10^, ^11^, ^12^, ^14^, ^30^, ^31^, ^32^, ^33^, ^34^, ^35^, ^36^, ^37^ ^]^ It should be noted most OSCs before 2015 were fullerene‐based acceptors (PC_71_BM, ICBA, etc.) with some limitations, such as the weak and narrow absorption and hardly tuned properties. Later, a new era of OSCs started with a fused‐ring nonfullerene acceptor (NFA) ITIC reported by Zhan et al., which gave an original PCE of 6.80%.^[^ ^38^ ^]^ The small‐molecule ITIC possesses an acceptor–donor–acceptor (A–D–A) backbone, in which the D and A units can be modified to finely tune absorptions, energy levels, and molecular packing properties. In the past 5 years, PCEs of 12–16% have been achieved in various NFAs cases via rational designing of the A and D unit chemical structures.^[^ ^1^, ^39^, ^40^, ^41^, ^42^, ^43^, ^44^, ^45^, ^46^, ^47^, ^48^ ^]^ Very recently, the efficiency has been pushed to ≈18% owing to the development of novel NFAs (Y6 and derivatives) and efficient polymer donors.^[^ ^49^ ^]^ Consequently, with the continuous development of efficient photoactive materials, significant progress has been achieved for flexible OSCs in recent years.^[^ ^21^, ^26^, ^27^, ^28^, ^50^, ^51^, ^52^, ^53^, ^54^, ^55^, ^56^, ^57^, ^58^, ^59^, ^60^, ^61^, ^62^, ^63^, ^64^ ^]^ As shown in **Table** **1**, the photovoltaic performances of representative flexible OSCs are summarized, and the molecular structures of corresponding materials are shown in **Figure** **2**.

**Table 1:** Summary of photovoltaic parameters and bending performance of representative flexible OSCs

FTE	Preparation method	Device structure	Active layer	*V*_oc_ [V]	*J*_sc_ [mA cm^−2^]	FF [%]	PCE [%]	Bending radius [mm]/Times	PCE^afterbending^ [%]	Efficiency maintenance [%]a)	Ref.
PET/ITO	Sputtering	Inverted	PTB7‐Th:IEICO‐4F	0.685	27.2	67.0	12.5	5/2000	11.25	90	[50]
Parylene/Su‐8/ITO	Sputtering	Inverted	PBDTTT‐OFT:IEICO‐4F:PC_71_BM	0.72	26.1	69.0	13.0	0.5/1000	12.61	97	[51]
PET/CNTs	Solution process	Conventional	PTB7:PC_71_BM	0.69	12.6	45.0	3.91	–	–	–	[52]
PI/Graphene	CVD	Conventional	PM6:Y6	0.84	25.8	70.2	15.2	2/1000	13.68	90	[53]
PET/PEDOT:PSS	Solution process	Conventional	P3HT:PC_71_BM	0.61	9.2	50.0	2.8	8/300	2.73	97.5	[54]
PET/PEDOT:PSS	Solution process	Conventional	PBDB‐T:IT‐M	0.93	15.5	70.3	10.12	5.6/1000	9.51	94	[26]
PET/PEDOT:PSS	Solution process	Conventional	PM6:Y6:PC_71_BM	0.828	23.6	72.0	14.06	7.5/500	13.88	98.7	[55]
PET/PEDOT:PSS	Solution process	Conventional	PM6:Y6	0.85	25.8	74.8	16.71	1.5/1000	15.8	96.1	[56]
PET/AgNWs	Solution process	Inverted	PTB7‐Th:PC_71_BM	0.76	17.4	64.2	8.75	1.5/1000	6.35	72.6	[57]
PET/AgNWs	Solution process	Inverted	PM6:IT‐4F	0.84	22.3	64.7	12.02	3/1000	7.21	60	[58]
PET/AgNWs (FlexAgNE)	Solution process	Inverted	PTB7‐Th:O6T‐4F:PC_71_BM	0.698	27.0	69.7	13.15	5/1000	12.63	96.0	[21]
			PBDB‐T:F‐M/PTB7‐Th:O6T‐4F:PC_71_BM	1.64	14.2	71.0	16.55	5/1000	15.78	95.3	
			PM6:BTP‐4F‐12	0.837	24.92	74.8	15.60	3/1000	14.20	91	[124]
								≈0/1000	12.95	83	
PEN/AgNWs	Solution process	Inverted	PM6:Y6	0.83	25.4	71.0	15.03	–	–	–	[59]
PET/AgNWs	Gravure printing	Inverted	PM6:Y6	0.83	25.1	73.6	15.28	–	–	–	[60]
PET/AgNWs/Al‐ZnO	Solution process	Inverted	PM6:Y6	0.830	25.1	73.0	15.21	4/1200	14.25	93.7	[27]
Treated cPI/AgNWs:PEDOT:PSS	Solution process	Conventional	PM6:Y6	0.83	26.0	70.0	15.12	–	–	–	[61]
PI:PS sphere/transferred AgNWs	Transferring	Inverted	PM6:N3:PC_71_BM	0.84	25.0	76.5	16.1	1/5000	13.69	85	[62]
PEI/Ag islands/PEDOT:PSS	Thermal evaporation/Solution process	Inverted	PTB7‐Th:PC_71_BM	0.79	17.3	74.0	9.9	–	–	–	[28]
PET/Ag mesh/PEDOT:PSS	Printing/Solution process	Conventional	PTB7:PC_71_BM	0.72	14.6	62.8	6.73	5/500	4.71	70	[63]
PET/Ag mesh/PH1000:AgNWs	Solution process	Inverted	PM6:IT‐4F	0.83	20.9	69.9	12.07	–	–	–	[64]

a) Efficiency maintenance (%) = PCE^after bending^/PCE^initial^ × 100%.

In this review, we present a systematic overview of the recent progress in flexible OSCs from the perspective of FTEs. The related FTEs (including ITO, carbon nanomaterials, conducting polymers, silver nanowires (AgNWs), and ultrathin metal films and metal meshes) used in flexible OSCs are sorted out. Meanwhile, a detailed discussion of the photovoltaic performances of flexible OSCs with various FTEs and photoactive materials is provided to unravel the underlying structure–property relationship. Finally, the opportunities and challenges of flexible OSCs are discussed.

## 2 Flexible OSCs Based on ITO

ITO (i.e., Sn‐doped In_2_O_3_), is a traditional transparent conductive film and typically used in optoelectronic devices due to the outstanding optoelectrical performance (sheet resistance ≈10 Ω sq^−1^ with transmittance >90%).^[^ ^65^ ^]^ However, the intrinsic brittleness of ITO is a significant drawback, which restricts its practical application in flexible devices.^[^ ^66^ ^]^ Moreover, ITO films are commonly prepared with physical vapor deposition (PVD) and magnetron sputtering methods, which are not compatible with the roll‐to‐roll process. Despite these issues, ITO is still widely used in flexible OSCs because it is among few commercial materials which predominate in the FTEs market for electronic devices, such as organic light‐emitting diodes, touch screens, and so on.

**Figure 3:**
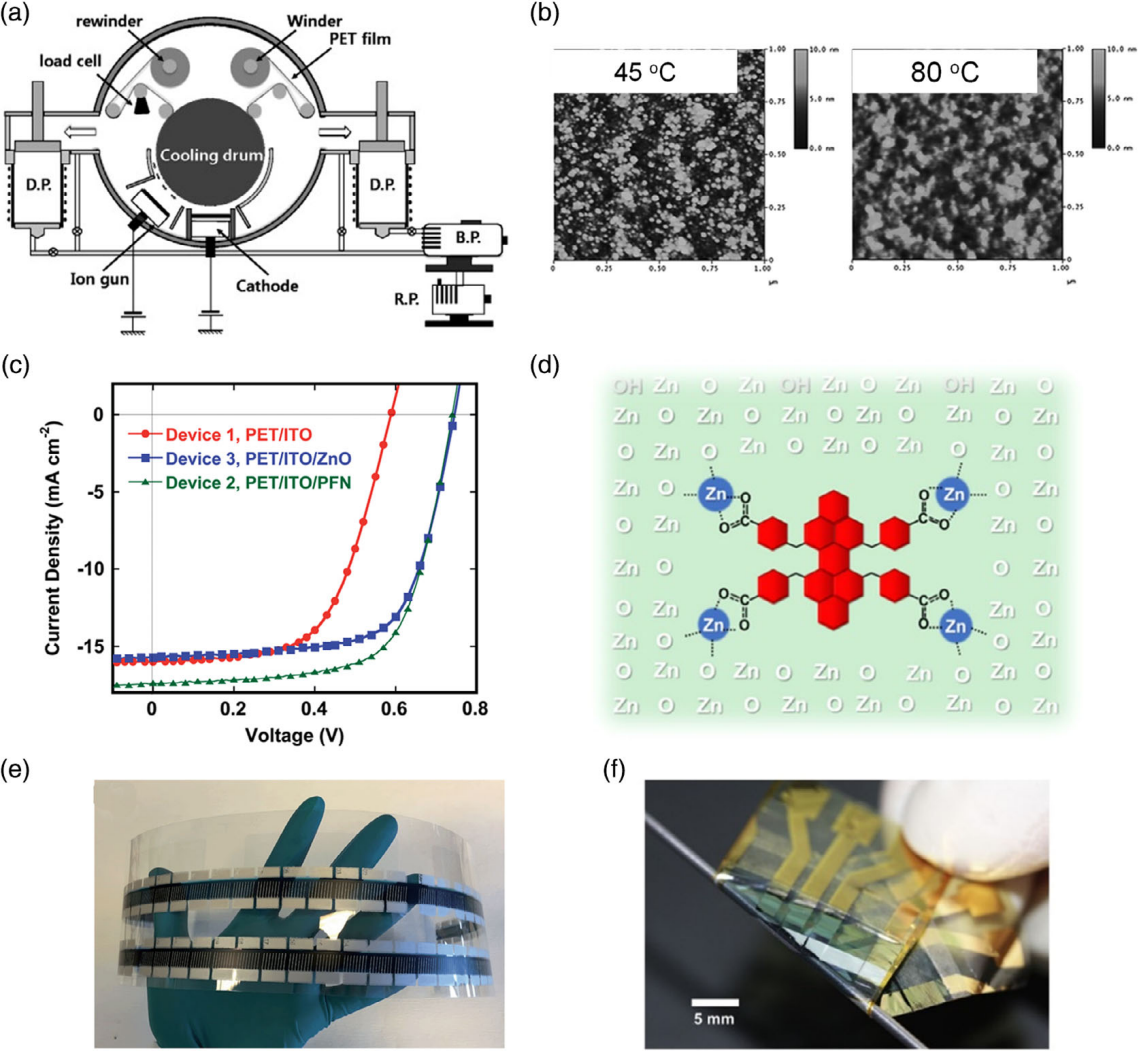
a) Schematic diagram of a specially designed roll‐to‐roll sputtering system. Reproduced with permission.^[^ ^68^ ^]^ Copyright 2009, Elsevier. b) Atomic force microscopy images of the 36 nm ZnO thin films grown on ITO‐coated PEN substrates at 45 and 80 °C. Reproduced with permission.^[^ ^69^ ^]^ Copyright 2010, The Royal Society of Chemistry. c) The *J–V* characteristics of the flexible PSCs with different device structures. Reproduced with permission.^[^ ^71^ ^]^ Copyright 2014, The Royal Society of Chemistry. d) The incorporation of PBI‐COOH into the ZnO lattice. Reproduced with permission.^[^ ^72^ ^]^ Copyright 2019, American Chemical Society. e) Long stripes of roll‐coated nonfullerene OSCs based on the flexible ITO substrate. Reproduced with permission.^[^ ^76^ ^]^ Copyright 2016, The Royal Society of Chemistry. f) Photograph of the ultraflexible OSC. The free‐standing device is wrapped over a syringe needle. Reproduced with permission.^[^ ^51^ ^]^ Copyright 2019, Elsevier.

In 2008, with flexible ITO electrodes, Jen's group reported inverted flexible OSCs with room‐temperature processed ZnO nanoparticles as electron transporting layer (ETL).^[^ ^67^ ^]^ Based on P3HT:PCBM blend, the flexible OSCs realized a lower PCE of 3.3% compared with the ITO‐based counterparts (3.6%), which is due to the lower transparency (80%) of the ITO‐coated plastic compared with ITO‐coated glass (88%), reducing the photon flux to the active layer, then leading to the lower short‐circuit current density (*J*_sc_). Subsequently, Kim et al. reported PET/ITO electrodes via continuous sputtering in a roll‐to‐roll sputtering system (**Figure** **3**a).^[^ ^68^ ^]^ However, the PET/ITO electrodes were prepared at room temperature, which provided a high sheet resistance of 47.4 Ω sq^−1^ with transmittance of 83.46%. Moreover, with a treatment of Ar ion beam on PET surface, an enhanced adhesion between PET substrate and ITO was achieved, leading to mechanically durable ITO electrodes with excellent robustness. A PCE of 1.88% was obtained for flexible OSCs with a conventional device structure. This provided a promising continuous sputtering method to produce low‐cost flexible OSCs with the roll‐to‐roll sputtering technique.

Electrode buffer layer between FTEs and active layer is also important for realizing highly efficient flexible OSCs. The interface layer can not only smooth FTEs surface but also ensure ohmic contact with active layers. As most commonly used ETL, ZnO offers high electron mobility, outstanding transmittance, low cost, and nontoxicity.^[^ ^50^, ^58^ ^]^ However, in rigid devices, ZnO layer is usually fabricated via a sol–gel process and requires annealing at high temperature (200 °C), which is not practical for roll‐to‐roll plastic substrates that only endure low temperature (<150 °C). To solve this problem, preparing ZnO layers at low temperature has aroused wide research interests. In 2010, Horng's group prepared ZnO layer on PEN/ITO at a low temperature of 80 °C via an atomic layer deposition process (Figure 3b).^[^ ^69^ ^]^ Using this PEN/ITO/ZnO, the inverted flexible OSCs with P3HT:PC_61_BM blend showed a higher PCE of 4.18% in comparison with the devices without ZnO layer (0.57%). The incensement in PCE can be ascribed to the fact that ZnO ETL can suppress hole diffusion and enhance electron collection of the PEN/ITO. Later, Ma et al. developed a microgravure roll‐to‐roll printing method to fabricate ZnO film at a low temperature (100 °C).^[^ ^70^ ^]^ Using the microgravure printed ZnO, the inverted OSCs with P3HT:PC_61_BM and PTB7‐Th:PC_71_BM blends yielded PCEs of 2.40% and 6.61%, respectively, which are comparable with those of spin‐coated ZnO counterparts. This method would promote the development of large‐area flexible OSCs via a microgravure roll‐to‐roll printing process.

To further improve the ETLs performance in flexible OSCs, developing novel materials to replace ZnO or doping ZnO films to improve its conductivity has been reported. In 2014, Wu et al. used an alcohol–water‐soluble conjugated semiconducting polymer, poly[(9,9‐bis(3′‐(*N*,*N*‐dimethylamino)propyl)‐2,7‐fluorene)‐alt‐2,7‐(9,9‐dioctylfluorene)] (PFN), to replace ZnO and prepare flexible OSCs at low temperature.^[^ ^71^ ^]^ With PTB7:PC_71_BM blend, the inverted flexible OSCs on PET/ITO/PFN achieved an impressive PCE of 8.71%, which is close to the rigid counterparts (9.21%). But its fill factor (FF) is relatively low (65.9% vs 69.99%), which can be further improved via interface engineering or novel high‐quality FTEs (Figure 3c). The simplicity of manufacturing flexible OSCs at room temperature can provide easy processability on large‐area devices. Recently, Xie's group reported a ZnO ETL doped with PBI‐COOH (four 4‐carboxyphenoxy substituents functionalized perylene bisimide) (called ZnO:PBI‐COOH; Figure 3d).^[^ ^72^ ^]^ It can be obtained at low annealing temperature (120–130 °C). With the organic dye dopant, the PBI‐COOH can bring about chemical bonding interaction, and further the improvement of ZnO crystallinity, thereby defects (e.g., oxygen vacancy and residual hydroxyl group) of ZnO layer can be effectively eliminated, leading to enhanced electron mobility. With ZnO:PBI‐COOH ETL, the inverted flexible OSCs with J71:ITIC blends yielded a PCE of 9.6%. Moreover, Zhou's group replaced ZnO with a polymer interfacial layer a‐PEIE (polyethyleneimine ethoxylated is processed from aqueous solution) for fabricating flexible OSCs, which was only processed at room temperature.^[^ ^50^ ^]^ With the protonated PEIE ETL, the inverted rigid OSC based on PCE‐10:IEICO‐4F blend delivered a PCE of 13.2%, even higher than the OSCs based on ZnO ETL (12.6%), arising from increased *J*_sc_ and FF. The higher *J*_sc_ is mainly ascribed to the higher transmittance of a‐PEIE compared with ZnO. In addition, the higher FF demonstrated that electrons can be efficiently collected by the a‐PEIE layer in NF OSCs after protonation. Thus, the room‐temperature a‐PEIE interface layer gave a highly efficient flexible OSCs (12.5%).

In addition, with the remarkable device performance of rigid OSCs exceeding the threshold for commercialization, the FTEs based on ITO were used in flexible OSCs from small‐area devices to large‐area modules. However, due to various complex reasons, PCEs have dropped dramatically from small‐area flexible OSCs to large‐area devices. First, because of the different coating processes involved, it is difficult to control the uniformity, thickness, crystallinity, and morphology of the film of a large‐area device compared with a small‐area device. Second, the performance of the large‐area OSC is still significantly limited by the effect of parasitic series resistance. This is because the power loss caused by the series resistance is proportional to the square of the device length. In addition, when implementing organic photovoltaic (OPV) modules, parasitic series resistance becomes a limiting factor, which forces the use of so‐called striped geometries. This method is widely used in OPV modules manufactured using a roll‐to‐roll process.^[^ ^73^, ^74^ ^]^ To solve the aforementioned issues, considerable efforts have been devoted to fabricating efficient large‐area devices. As early as 2010, Krebs's group reported large‐area flexible OSCs via a full roll‐to‐roll solution process.^[^ ^75^ ^]^ With P3HT:PCBM blend, the large module (360 cm^2^) achieved a PCE of 1.69%. Later, Zhan et al. designed large‐area flexible OSCs based on NFAs with all fabrication procedures in ambient atmosphere.^[^ ^76^ ^]^ Based on PTB7‐Th:IEIC active layer, flexible devices exhibited PCEs up to 2.26% with active area of 0.66 cm^2^ (Figure 3e). It is pointed out that this offers the first example for flexible OSCs based on NFAs with PCEs exceeding 2% via an all roll‐coating process. More importantly, the NF OSCs offered better stability than fullerene‐based counterparts. However, for large‐area devices, it is difficult to achieve high PCEs as compared with small‐area devices. The reason for this is complex, for example, the coating processes, spin and slot‐die coating, are different, leading to different uniformity, thickness, crystallization, and morphology of the corresponding films. Recently, Chen's group explored the morphological evolution mechanism of active layer via various coating/slot‐die printing methods.^[^ ^20^ ^]^ A strategy of applying the shear impulse during the coating/printing process was presented by them, and with this method, the film morphology evolution was well controlled in large‐area devices. Thus, with active area of 1.04 cm^2^, the flexible OSCs with PBDB‐T:ITIC blend exhibited a high PCE of 9.77% via a slot‐die printing. Moreover, 15 cm^2^ flexible modules achieved a PCE of 8.90%. A possible reason for PCE decrease of large‐area devices is the enhancement of electrical and optical losses, which is a decisive parameter in determining the device performance. This view was further confirmed by Wei et al.^[^ ^74^ ^]^ With PET/ITO electrode, a PCE of 7.44% is obtained for device (1 cm^2^) with PTB7‐Th:COi8DFIC:PC_71_BM blend, which is far lower than that of small‐area devices. To further improve FTEs conductivity, PET/silver grid electrodes were adopted in the flexible devices. Compared with PET/ITO‐based devices, a remarkable higher PCE was obtained for PET/silver grid‐based devices, arising from the increased *J*_sc_ and FF. The higher *J*_sc_ is ascribed to the higher transmittance of the PET/silver grid compared with PET/ITO. Meanwhile, the lower resistance of PET/silver grid electrodes in comparison with PET/ITO (1.5 vs 30 Ω sq^−1^) leads to an increased FF. Finally, using PET/silver grid electrodes, the slot‐die‐coated flexible devices (1 cm^2^) exhibited a high PCE of 12.16% in comparison with that of the spin‐coated rigid devices with small area of 0.04 cm^2^ (12.37%). Moreover, an outstanding PCE of 10.09% was obtained for the large‐area module (25 cm^2^), which demonstrated the minor PCE losses in large‐scale flexible OSCs.

Furthermore, ultraflexible organic semiconductor devices with integration of multifunctionalities are an important goal in organic electronic field. Among them, the ultraflexible OSCs have also been widely studied. In recent years, Someya et al. fabricated 3 μm‐thick ultraflexible OSCs using perylene/Su‐8/ITO electrodes. A high PCE of 13% was obtained with PBDTTT‐OFT:IEICO‐4F:PC_71_BM blend, and the PCE retained 12.61% after 1000 cycles bending (radius = 0.5 mm).^[^ ^51^ ^]^ In addition, stretchable and waterproof ultraflexible OSCs have also made remarkable progress in recent years.^[^ ^77^, ^78^ ^]^ Thus, a lot of efforts should be devoted in developing FTEs with ultrathin substrate to fabricate ultraflexible and integrated multifunctional organic optoelectronic devices for practical applications. In addition, using all‐polymer active layers may be another strategy to realize superior mechanical stability for flexible devices.^[^ ^79^ ^]^ Kim et al. reported mechanically robust all‐polymer OSCs with polymer donor and polymer acceptor (PBDTTTPD:P(NDI2HD‐T)).^[^ ^80^ ^]^ Compared with the polymer/PCBM‐based devices, the all‐polymer devices exhibited significantly enhanced strength, with 60‐ and 470‐fold enhancements in elongation at break. The remarkable mechanical properties of all‐polymer OSC make it a promising candidate for flexible and portable devices.

Even though the ITO‐based FTE has been successfully applied in flexible OSCs, some significant drawbacks make ITO not the ideal choice for high‐quality FTEs. The high cost due to shortage of indium and the intrinsic brittle feature of ITO greatly limited its future application in flexible electronics. Therefore, exploring novel FTE materials with combined good features of low resistance, high transmittance, excellent mechanical durability, and smooth surface becomes more and more urgent for flexible OSCs.

## 3 Flexible OSCs Based on Carbon Nanomaterials

Carbon nanomaterials, such as graphene and carbon nanotubes (CNTs), have been extensively utilized in flexible OSCs. This is because of their merits of abundance, high conductivity, good transmittance, and excellent flexibility. In the following section, their application in the flexible OSCs will be introduced briefly.

### 3.1 Graphene

Since Geim and coworkers first prepared 2D graphene films with a facile mechanical exfoliation (repeated peeling) process, research on graphene has been a hot topic.^[^ ^81^ ^]^ Graphene is composed of 2D *sp* ^2^‐hybridized carbon atoms arranged in a honeycomb lattice. Graphene has become a popular FTE material in flexible OSCs owing to its intrinsic mechanical flexibility, high thermal and chemical stability, and low contact resistance with the organic materials.^[^ ^82^ ^]^ There are several methods for manufacturing graphene thin films, including mechanical exfoliation, chemical vapor deposition (CVD), reduction of graphene oxide (rGO), and so on.^[^ ^83^ ^]^ High‐quality graphene films can be obtained by mechanical exfoliation method. However, this method is not compatible with large‐scale production, commonly used in lab research. In recent years, rGO, CVD, and other solution‐based methods have been extensively studied.

#### 3.1.1 rGO Method

**Figure 4:**
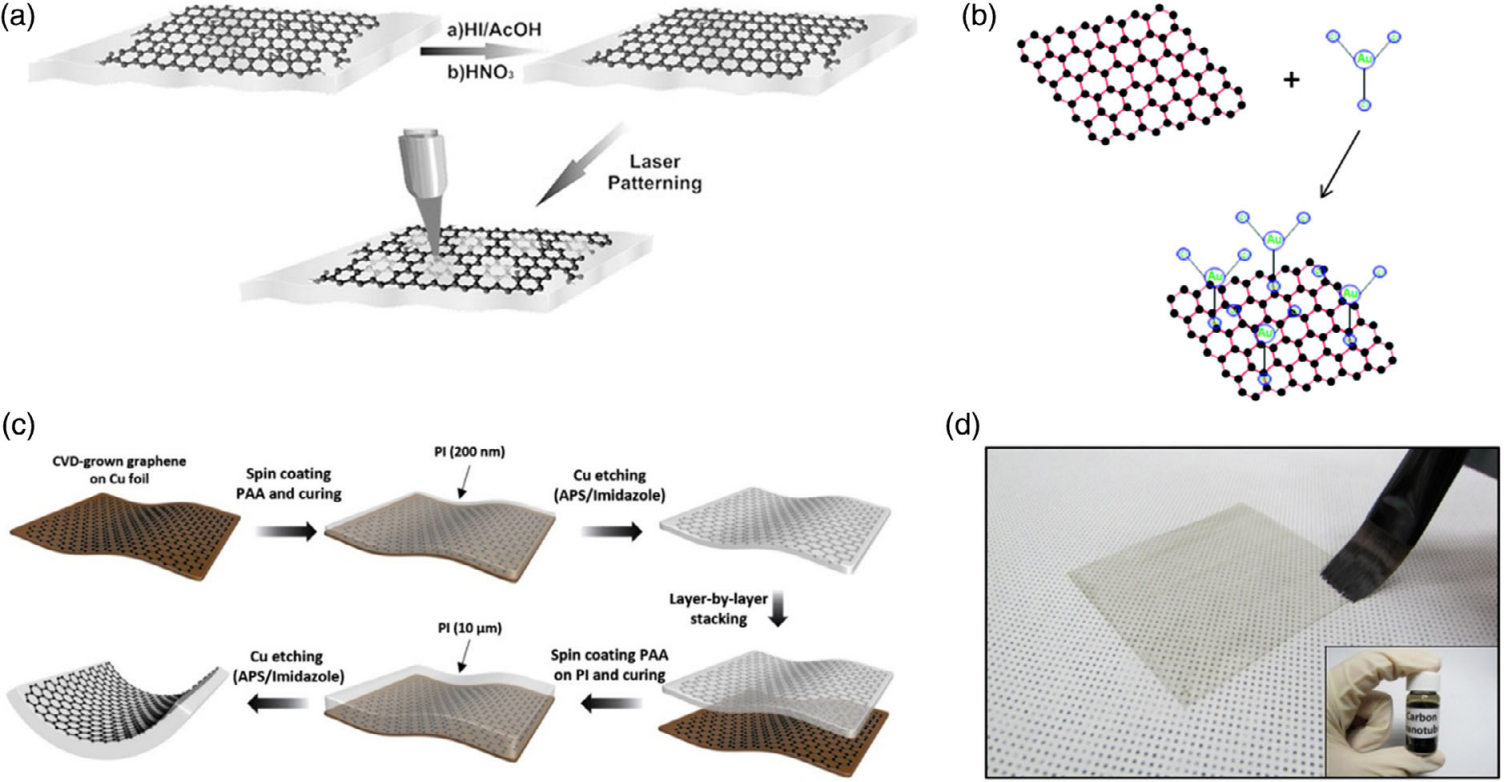
a) Schematic illustration of the rGO mesh electrodes preparation. Reproduced with permission.^[^ ^86^ ^]^ Copyright 2015, Wiley‐VCH. b) The doping process illustration. Reproduced with permission.^[^ ^90^ ^]^ Copyright 2015, The Royal Society of Chemistry. c) Schematic describing the two‐step fabrication process of PI@GR. Reproduced with permission.^[^ ^53^ ^]^ Copyright 2020, Elsevier. d) Picture of brush printing process (upper panels) to fabricate transparent CNT network electrodes and transparent 3‐brush CNT electrode. Reproduced with permission.^[^ ^147^ ^]^ Copyright 2013, Elsevier.

The rGO is an extensively used method to prepare graphene films owing to its low cost and solution processability. Also, the use of rGO as FTE in flexible OSCs has also attracted much research interests. In 2010, Zhang and coworkers transferred rGO onto PET substrates.^[^ ^84^ ^]^ The rGO film (16 nm) showed a high sheet resistance of 3.2 kΩ sq^−1^ with transmittance of 65%. With P3HT:PC_61_BM blend, the flexible OSCs exhibited a PCE of 0.78%, and the PCE retained 2.9% after 1000 cycles bending (radius = 5 mm). These results indicated the excellent mechanical durability of rGO films, revealing their potential in flexible electronics. However, the high‐temperature GO‐reduction process makes it unfit to the plastic substrates. To address this issue, various reduction methods have been conducted in low temperature. In 2013, Kymakis et al. presented a GO films reduction method via a pulsed femtosecond laser beam (LrGO), and no thermal annealing and transferring is required.^[^ ^85^ ^]^ The LrGO film with the thickness of 16.4 nm exhibited a high resistance of 1.6 kΩ sq^−1^ with transmittance of 70%. With P3HT:PC_61_BM blend, the flexible OSCs based on LrGO electrodes got a PCE of 1.1%, and retained function even for the bending angle as high as 135°. Later, the same group created rGO micromesh (rGOMM) electrodes via a laser‐based patterning technique (**Figure** **4**a).^[^ ^86^ ^]^ The rGOMM electrodes offered a sheet resistance of 565 Ω sq^−1^ with transmittance of 59%. With PCDTBT:PC_71_BM blend, the flexible OSCs based on rGOMM electrodes got a PCE of 3.05%, higher than the devices based on pristine rGO (1.78%), which is mainly ascribed to the enhanced optoelectrical properties of rGOMM electrodes.

Low‐cost rGO films have been widely used in flexible OSCs. However, due to the defects and low conductivity of rGO films, the device performance is still limited. Functional modification of rGO films may be considered to reduce defects and improve optoelectrical performance for high‐quality rGO films design.

#### 3.1.2 CVD Method

CVD is considered to be one of the most promising methods to fabricate high‐performance graphene films with smooth surface and high conductivity. In 2010, Zhou's group reported a continuous and highly flexible graphene‐based FTE via a CVD method.^[^ ^87^ ^]^ The graphene electrode exhibited low surface roughness and sheet resistance of 230 Ω sq^−1^ with transmittance of 72%. Using the copper phthalocyanine (CuPc)/C60 active layer, the flexible OSCs based on CVD graphene electrodes obtained a PCE of 1.18%, similar to the devices based on PET/ITO (1.27%).

To further reduce the resistance of CVD graphene films, multilayer stacking and doping as efficient methods have been demonstrated. In 2012, Li and coworkers prepared a sandwiched GO/tetracyanoquinodimethane (TCNQ)/GO electrode through a layer‐by‐layer doping method.^[^ ^88^ ^]^ The multilayered GO/TCNQ/GO electrodes showed resistance of 278 Ω sq^−1^ with high transmittance of 92.2%. Using P3HT:PCBM blend, the flexible OSCs yielded a PCE of 2.58%. Later, Lee et al. prepared a HNO_3_ or SOCl_2_‐doped multilayer graphene films via CVD growth.^[^ ^89^ ^]^ After doping, the sheet resistance dramatically decreased from 850 to 450 Ω sq^−1^; meanwhile, almost unchanged transmittance of 90% was obtained. With P3HT:PCBM blend, the doped MLG electrodes‐based flexible OSCs got a PCE of 2.54%, higher than the pristine MLG‐based devices (1.77%), attributing to the enhanced conductivity. Moreover, PCEs of the flexible OSCs could still sustain 2.5% even with a radius of 5.2 nm. In 2015, Jang's group reported a single‐layer graphene nanoribbon with doping of Au as a FTE in flexible tandem OSCs (Figure 4b).^[^ ^90^ ^]^ This electrode showed sheet resistance of 221 Ω sq^−1^ with transmittance of 97%. With the SMPV1:PC_71_BM and PTTBDT‐FTT:PC_71_BM as the front and rear cells, the flexible tandem OSCs showed a high PCE of 8.48%. In addition, Park et al. reported multilayer stacking graphene electrodes with a low pressure CVD (LPCVD) method.^[^ ^91^ ^]^ The LPCVD graphene electrodes offered sheet resistance of 300 Ω sq^−1^ and high transmittance of 92%. Using PTB7:PC_71_BM as the active layer, the conventional and inverted flexible OSCs achieved PCEs of 6.1% and 7.1%, respectively. Recently, Koo et al. developed a three‐layer stacked graphene electrodes through direct integration of graphene and PI (PI@graphene) (Figure 4c).^[^ ^53^ ^]^ The PI@graphene electrodes offered clean surface with sheet resistance of 83 Ω sq^−1^ and high transmittance of 92%. With PM6:Y6 blend, flexible OSCs based on PI@graphene electrodes showed a record‐high PCE of 15.2%, close to ITO‐based rigid counterparts (15.7%). Furthermore, a high retention (90%) of the initial PCE was obtained after 1000 cycles bending (radius = 2 mm), indicating the excellent bending performance of the PI@graphene electrodes. The excellent PCE of over 15.2% has been achieved for CVD‐grown graphene‐based flexible OSCs, indicating the distinct merits of this method.^[^ ^53^ ^]^ However, there are also some drawbacks for CVD method, such as high cost and the film transferring fabrication method, limiting its large‐scale production. Thus, it is critical to develop low‐cost and easy technologies of graphene preparations.

### 3.2 Carbon Nanotubes

In 1991, Iijima first reported a finite carbon structure with needle‐like tubes (CNTs), which is a hollow cylinder winded with planar graphene sheets, including single‐walled CNTs (SWCNTs) and multiwalled CNTs (MWCNTs).^[^ ^92^ ^]^ In recent years, CNTs have also shown great potential as FTE materials due to its intrinsic good electrical properties, mechanical flexibility, high transparency, and solution treatability at room temperature.^[^ ^24^ ^]^

In 2006, McGehee's group prepared PET/SWCNTs electrodes via a transfer printing process.^[^ ^93^ ^]^ The flexible SWCNTs electrodes showed low roughness with resistance of 200 Ω sq^−1^ and high transmittance of 85%. Based on the active layer of P3HT:PC_61_BM, flexible OSCs showed a PCE 2.5%, which was close to the rigid ITO devices (3.0%). In addition, the PET/SWCNTs‐based devices exhibited no efficiency degradation after bending at a radius of 5 mm, implying excellent mechanical stability of the PET/SWNT electrodes. As an important factor for large‐scale application of CNTs in flexible OSCs, its fabrication method demands low cost and facile process. Except for the common dry deposition and solution coating, some new methods, such as brush‐painting, have been proposed by Kim and coworkers (Figure 4d).^[^ ^94^ ^]^ The brush‐painted PET/CNTs electrodes showed low surface roughness, resistance of 286 Ω sq^−1^ with transmittance of 78.45%. With P3HT:PC_61_BM blend, the CNTs‐based flexible OSCs got a PCE of 1.63%, implying the potential of brush‐painted CNTs films as low‐cost FTEs for large‐area flexible OSCs.

To improve the performance of pristine CNTs, lots of efforts have been devoted to CNTs electrodes, such as modification or hybridization. In 2012, Zarbin's group reported polyanilines (PANI):CNTs composites electrodes.^[^ ^95^ ^]^ PANI acted as conductive glue between CNTs, leading to improved conductivity. The PANI:CNTs films exhibited dramatically decreased resistance from 85 000 to 295 Ω sq^−1^, while the transmittance retained almost unchanged (89%). Due to the unfold coil‐like structure, the increasing delocalized carrier transportation of PANI:CNTs films led to high conductivity. With the active layer of F8T2/C60, flexible OSCs yielded a PCE of 2.27%, which is better than the corresponding ITO counterparts. Subsequently, Matsuo and coworkers developed SWCNT films doped with MoO_*x*_.^[^ ^96^ ^]^ After doping, the sheet resistance of SWCNT films was significantly decreased. With PTB7:PC_71_BM blend, the flexible OSCs obtained a PCE of 3.91% and offered almost unchanged PCE after 10 times bending (radius = 5 mm).

As mentioned earlier, graphene and CNTs have demonstrated as good candidates for FTEs and been extensively used in flexible OSC devices. However, there are still some issues that need to be solved. The high cost of CVD‐grown graphene and CNTs is a critical bottleneck for large‐scale production, which the researchers should place value on. In addition, the optoelectrical properties of graphene and CNTs need to be further optimized to improve the device performance. Graphene and CNTs as carbon nanomaterials are suitable for chemical functionalization. Thus, chemical grafting using the conductive carbon quantum dots or alcohol‐soluble polymers may be considered to modify the defects of graphene and CNTs. In addition, exploring the emerging 2D conductive materials (such as MXene and graphdiyne) may be an effective strategy to improve their optoelectrical properties. Finally, developing new low‐cost and easy methods of graphene and CNTs preparations, such as printing technology, is essential for large‐area manufacturing and device application.

## 4 Flexible OSCs Based on Conducting Polymers

Conducting polymer is another promising alternative material to replace ITO. The most representative conducting polymers in FTEs is poly(3,4‐ethylenedioxythiophene):poly(styrene sulfonate (PEDOT:PSS), offering intrinsic flexibility and easy processing. PEDOT:PSS is an aqueous solution, including two ionomers: conjugated PEDOT (polycation) and PSS (polyanion). However, the electrical property of PEDOT:PSS is not good enough in comparison with ITO. Thus, it is a primary concern for PEDOT:PSS‐based electrodes to improve the conductivity. Many factors determine the conductivity, such as phase separated morphology of PEDOT and PSS, crystallization of conducting PEDOT, removal of insulating PSS, and so on. Considering the PSS has an insulating nature, the conductivity could decrease when reducing the PSS ratio. Heraeus has prepared various PEDOT:PSS with multiple PSS ratio. Clevios PVP AI 4083 (PEDOT:PSS = 1:6) provides conductivity ranging from 10^−3^ to 10^−4^ S cm^−1^, and is widely applied in OSCs as hole transporting material (HTL). In addition, other grades of PEDOT:PSS have been reported, including PH500, PH510, and PH1000 (PEDOT:PSS = 1:2.5), with conductivity ranging from 0.2 to 1 S cm^−1^.

**Figure 5:**
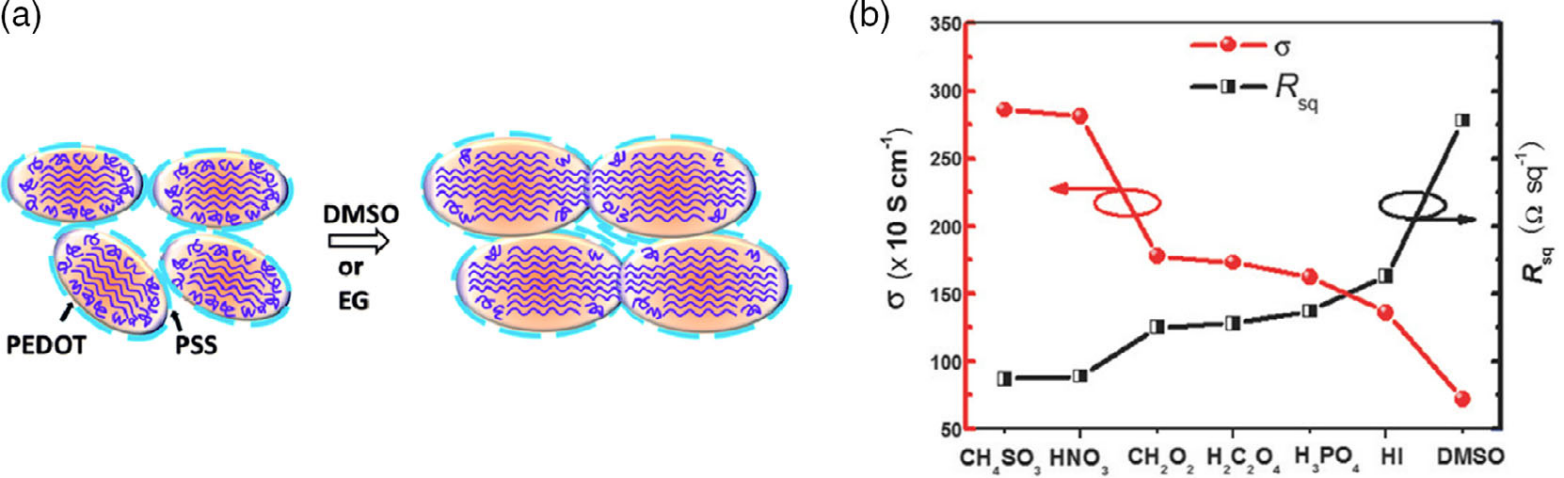
a) Schematic model of structural modification in PEDOT:PSS with the addition of DMSO and EG. Reproduced with permission.^[^ ^104^ ^]^ Copyright 2014, The Royal Society of Chemistry. b) Comparison of square resistance and electrical conductivity of the PEDOT:PSS with diverse acid treatments at room‐temperature and DMSO doping. Reproduced with permission.^[^ ^26^ ^]^ Copyright 2018, Wiley‐VCH.

In 2002, the first example of OSCs using PEDOT:PSS and its derivatives as FTEs was reported by Inganäs's group, which proved that it was feasible to use solution processed conductive polymers instead of ITO to fabricate OSCs.^[^ ^97^ ^]^ In 2004, Aernouts and coworkers used PEDOT:PSS to fabricate flexible OSCs;^[^ ^98^ ^]^ the OSCs with the PEDOT:PSS‐based FTEs showed the same open‐circuit voltage (*V*_oc_) but a lower short‐circuit current (*I*_sc_) compared with the ITO counterparts (2.4 × 10^−4^ vs 9.3 × 10^−4^ A). Such a change could be ascribed to the difference in the conductivity of PEDOT:PSS in comparison with ITO (400 vs 50 Ω sq^−1^). It is clear that the performance of flexible devices is severely restricted by the low conductivity of PEDOT:PSS.

Several strategies have been developed to improve the PEDOT:PSS conductivity, such as postsurface treatment (acids, etc.) and doping PEDOT:PSS with some additives, including dimethyl sulfoxide (DMSO), ethylene glycol (EG), *N*,*N*‐dimethylformamide (DMF), sorbitol, and glycerol.^[^ ^99^, ^100^, ^101^, ^102^, ^103^, ^104^ ^]^ In 2008, Kim's group reported efﬁcient flexible OSCs using 5% DMSO‐doped PH500 as highly conductive electrodes.^[^ ^54^ ^]^ After doping, the DMSO‐doped PH500 electrodes exhibited an enhanced conductivity from <1 to 550 S cm^−1^. The flexible OSC with P3HT:PCBM blend obtained a PCE of 2.8%, very close to that of the PET/ITO‐based devices (2.9%). Moreover, the devices based on DMSO‐doped PH500 exhibited superior mechanical robustness, of which efficiency maintained well during 300 cycles bending with a radius of 8 mm. In addition, Zhou and coworkers prepared a bilayer conducting polymer FTE with the composition of PH500 and PEDOT:PSS 4083 (Baytron PVP AI).^[^ ^105^ ^]^ Based on APFO‐3:PCBM active layer, the PCE of the flexible OSC is 2.2%, which is equivalent to 80% of the PCE of the rigid ITO counterparts. The simple and effective preparation method of bilayer FTEs and the comparable performance of flexible devices made bilayer processable PEDOT:PSS an alternative to ITO for flexible OSCs. In 2011, Bao's group improved the PEDOT:PSS conductivity via doping with a blend of DMSO and fluorosurfactant (Zonyl‐FS300).^[^ ^25^ ^]^ It is pointed out that the Zonyl‐FS300 could improve the PEDOT:PSS wetting properties, making PEDOT:PSS deposited easily on hydrophobic substrates. The doped‐PEDOT:PSS electrodes achieved an outstanding optoelectrical performance (transmittance 82% with sheet resistance 46 Ω sq^−1^). Finally, using P3HT:PCBM blend, the flexible OSCs based on PDMS/doped‐PEDOT:PSS electrodes showed a comparable performance to that of the ITO‐based counterparts. In 2012, Bauer and coworkers prepared ultrathin PET/PEDOT:PSS electrodes using the similar doping treatment (DMSO/Zonyl FS‐300).^[^ ^106^ ^]^ An equal PCE to the rigid counterparts was achieved for flexible OSCs with this electrode, while the total thickness was less than 2 μm. It is pointed out that the specific weight of flexible OSCs could be as large as 10 W g^−1^ and could sustain the harsh mechanical deformation. To understand the mechanism for conductivity changes after doping a morphological model was provided to explain the influence of additives in PEDOT:PSS.^[^ ^104^ ^]^ The additives modified the internal crystalline ordering of PEDOT nanocrystals, leading to increased crystal size, highly crystalline nanofibrils, and also rearrangement of PSS (**Figure** **5**a). These results indicate the enhancement of PEDOT:PSS conductivity and OSCs performance.

Posttreatment was also studied to improve the PEDOT:PSS conductivity. In 2012, Ouyang's group first reported PEDOT:PSS films with treating of dilute sulfuric acids (H_2_SO_4_), and its conductivity was greatly enhanced from 0.3 to 3065 S cm^−1^.^[^ ^101^ ^]^ A transmittance of 80% with sheet resistance 39 Ω sq^−1^ was obtained for the PEDOT:PSS films with the treatment of H_2_SO_4_. Later, Lee et al. exposed PEDOT:PSS films to H_2_SO_4_ fume and achieved high conductivity (4380 S cm^−1^).^[^ ^107^ ^]^ The PEDOT:PSS film exhibited resistance of 46 Ω sq^−1^ with high transmittance of 90%. The flexible OSCs achieved similar device performances with ITO and H_2_SO_4_‐treated PEDOT:PSS as the electrodes. This low sheet resistance might be attributed to protonation effect of PSS, which forms closely packed nanocrystals.

Indeed, H_2_SO_4_ can effectively improve the PEDOT:PSS conductivity; however, the strong acid could cause damage to the plastic substrates, even leading to environmental issues. To solve the problem, various weak acids, including sulfurous acid, acetic acid, phosphoric acid, and so on, have been applied in PEDOT:PSS films.^[^ ^108^ ^]^ Ouyang and coworkers treated PEDOT:PSS films with the methane sulfonic acid (CH_3_SO_3_H), a mild and weak acid, and achieved an enhanced conductivity from 0.3 to 3300 S cm^−1^.^[^ ^109^ ^]^ Fan et al. prepared high‐quality PET/PEDOT:PSS electrodes with the treating of methanol and CH_3_SO_3_H, and the conductivity was increased to 3560 S cm^−1^.^[^ ^110^ ^]^ Using this electrode, flexible OSCs with P3HT:PCBM blend achieved a PCE of 3.92%, close to the rigid devices based on ITO electrodes (4.30%). Later, Yan and coworkers used a mild acid treatment, including two steps, dipping with the CH_4_SO_3_ and then soaking with the phosphoric acid, combined with a transfer‐printing technology to prepare PDMS/PEDOT:PSS electrodes.^[^ ^111^ ^]^ The conductivity was improved to 3560 S cm^−1^. Due to the less residues of the mild and weak acids in PEDOT:PSS matrix, flexible OSCs showed a higher PCE and better stability than the H_2_SO_4_‐treated PEDOT:PSS‐based devices. Finally, with PBDTT‐S‐TT:PC_71_BM blend, flexible devices obtained a PCE of 5.38%. Ge's group fabricated PEDOT:PSS on PET substrates with the CH_4_SO_3_ treating at room temperature and got a high conductivity of 2860 S cm^−1^ (Figure 5b).^[^ ^26^ ^]^ Using PBDB‐T:IT‐M blend, flexible OSCs obtained a PCE of 10.12%, close to the ITO rigid counterparts (11.01%). These outstanding performances are attributed to the removal of insulating PSS and further a better phase separation of the PEDOT and PSS. With these, the PCE sustained 94% of the initial efficiency during 1000 times bending (radius = 5.6 mm). Subsequently, based on PM6:Y6 active layer, flexible OSCs using this electrode yield a high PCE of 14.20%, indicating a simple room‐temperature method for fabricating high‐quality FTEs in flexible OSCs. Recently, Fan's group prepared PET/PEDOT:PSS via spray doping with a tiny perchloric acid (HClO_4_), and obtained a low resistance of ≈29 Ω sq^−1^.^[^ ^56^ ^]^ Flexible OSCs with PM6:Y6 blend yielded an excellent PCE of 16.71%. The outstanding performance was attributed to the spray doping of HClO_4_, which can pull down the Fermi level of the anode, reduce the sheet resistance, and lead to a close contact on interface. Moreover, the flexible devices retained 93.6% of the initial efficiency after 1000 times harsh bending (radius = 1.5 mm), indicating the excellent mechanical stability. Furthermore, the first all‐plastic full‐solution‐processed tandem OSCs were achieved by Zhou's group with PEDOT:PSS‐based FTE.^[^ ^112^ ^]^ The conductivity of the charge recombination layer (PH1000/AI 4083) was precisely adjusted and achieved a conductivity of 10^−2^ S cm^−1^. Multijunction solar cells (up to seven junctions) with all solution‐treated layers have been prepared, exhibiting the maximum *V*_oc_ of 5.37 V. These results indicate the great potential of CRL in large‐area multijunction OSCs.

Therefore, it has been demonstrated that the FTEs based on conducting polymer, such as PEDOT:PSS, were expected to replace ITO in flexible OSC devices. However, the flexible OSCs based on PEDOT:PSS electrodes suffered from degradation due to acidity and hydrophilicity. In addition, the optoelectrical properties of the PEDOT:PSS film are not high enough. To design FTE materials with better optoelectrical properties, postsurface treatment (mild acids, etc.) and doping are suggested to tune crystalline ordering and increase crystal size of PEDOT. It is also useful to reduce the insulated PSS by water washing and other methods which can remove the PSS and modify phase separated morphology of PEDOT and PSS. Finally, rational design of new conducting polymers with high conductivity and stability may be another strategy to achieve high‐quality conducting polymers.

## 5 Flexible OSCs Based on AgNWs

As alternatives to ITO, metal nanowires, particularly AgNWs, exhibit significant promise as effective highly conductive FTE due to its combined merits including superior optoelectrical properties and good mechanical flexibility. More importantly, the solution‐processed AgNWs are applicable for roll‐to‐roll production in large‐area devices.

**Figure 6:**
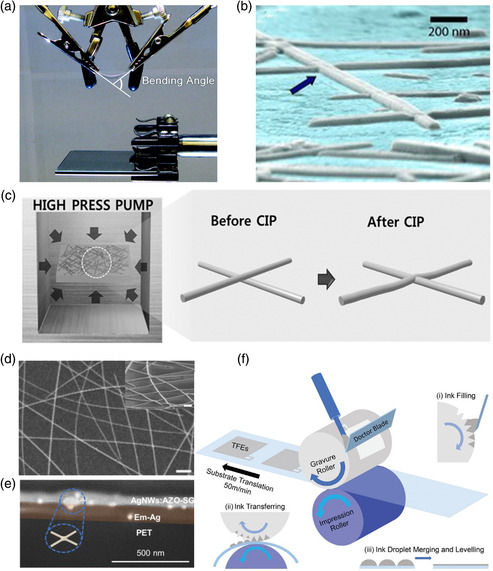
a) The experimental setup used for measuring the *J*–*V* curves of flexible devices. Reproduced with permission.^[^ ^114^ ^]^ Copyright 2011, American Chemical Society. b) Close‐up angled surface showing junctions of nanowires embedded into the polymer (indicated by arrow). Reproduced with permission.^[^ ^123^ ^]^ Copyright 2011, Wiley‐VCH. c) Schematic of the fabrication of an AgNW electrode using CIP for AgNW junctions on a suspended plastic substrate in water. Reproduced with permission.^[^ ^57^ ^]^ Copyright 2017, Wiley‐VCH. d) Surface and cross‐sectional scanning electron microscopy (SEM) images of FlexAgNEs, scale bars: 500 and 200 nm (inset). Reproduced with permission.^[^ ^21^ ^]^ Copyright 2019, Springer Nature. e) Cross‐sectional SEM images of the Em‐Ag/AgNWs:AZO‐SG. Reproduced with permission.^[^ ^27^ ^]^ Copyright 2020, Wiley‐VCH. f) Schematic diagram of the high‐speed gravure printing process used to print AgNW electrodes. Insets: i) the doctor blade forces the ink to fill the gravure cavities; ii) the ink is transferred from the cavities of the gravure roller to the substrate; and iii) ink leveling on the substrate. Reproduced with permission.^[^ ^60^ ^]^ Copyright 2020, Wiley‐VCH.

In 2008, Peumans's group first reported AgNWs electrodes in flexible OSCs. The AgNWs‐based FTEs are prepared entirely from solution and require only a low‐temperature annealing.^[^ ^113^ ^]^ Based on AgNWs/PEDOT:PSS composite electrodes, the flexible devices using deposited active bilayer (CuPc/PTCBI) showed a 19% higher photocurrent compared with the ITO rigid counterparts. In 2011, You and coworkers fabricated highly conductive PET/AgNWs electrodes via a spraying process.^[^ ^114^ ^]^ With a conventional device structure, the flexible OSCs based on PBnDT‐DTffBT:PC_61_BM blend got a PCE of 2.5%. The relatively low PCE was caused by the low WF and bad ohmic contact with the active layer for the PET/AgNWs electrodes. More importantly, the flexible OSCs retained PCE of 2.3% after ten cycles bending even for bending angle up to 120° with a 7.2 mm bending radius (**Figure** **6**a). Subsequently, Pei's group reported AgNWs–polymethacrylate composite electrodes (AgNW‐SL), consisting of a stack of AgNWs with long and short length.^[^ ^115^ ^]^ This FTE offered transmittance of 80% with resistance of 10 Ω sq^−1^. The flexible OSCs with P3HT:PCBM blend showed a PCE of 3.28%, similar to the ITO‐based rigid devices (3.34%). These outstanding performances could be attributed to the high‐quality AgNW‐SL electrodes, which indicated that AgNWs‐based FTE is a promising candidate in flexible OSCs.

Despite the success that has been achieved in flexible OSCs using AgNWs electrodes, there are still some critical challenges, such as constructing network structure, decreasing contact resistance, and reducing surface roughness. To address these issues, several strategies have been used, such as doping, posttreatment (thermal annealing, pressing, etc.), and forming a protective layer, including polymer, graphene, and metal oxide.^[^ ^21^, ^57^, ^88^, ^115^, ^116^, ^117^, ^118^, ^119^, ^120^, ^121^, ^122^ ^]^ In 2011, Peumans’ group fabricated composite FTEs via embedding AgNWs into PEDOT:PSS films, and the most important feature of these FTEs is low roughness (Figure 6b).^[^ ^123^ ^]^ With these, the composite FTE can tolerate >5 times larger mechanical strain than ITO, suggesting its superior mechanical flexibility. With P3HT:PCBM blend, flexible OSCs exhibited a PCE of 3.8%, similar to the rigid devices based on ITO (4.2%). Later, Seo et al. created close‐contact junctions of AgNWs via a facile cold isostatic pressing (CIP) method at room temperature, as shown in Figure 6c. This CIP method can minimize the surface roughness, increase the contact of AgNWs, and further enhance the conductivity of FTEs.^[^ ^57^ ^]^ With PTB7‐Th:PC_71_BM blend, flexible OSCs based on CIP‐processed AgNWs electrodes yielded a PCE of 8.75% and presented a high retention (95.5%) of the initial efficiency after 1000 cycles bending (radius = 4.8 mm). More importantly, this FTE allows 100% manufacturing yield of flexible OSCs. In 2019, Chen and coworkers created an effective but facile strategy to fabricate AgNWs electrodes (named FlexAgNEs) via ionic electrostatic charge repulsion. A grid‐like AgNWs network was obtained via spin coating (Figure 6d).^[^ ^21^ ^]^ The FlexAgNEs offers combined good features simultaneously, including high conductivity (≈10 Ω sq^−1^), superior transmittance (≈92%, excluding the substrate), and low surface roughness (8.2 nm). To demonstrate the FlexAgNEs universality, various types of donors and acceptors were used to fabricate flexible OSCs. High efficiencies of 13.15% and 16.55% for single‐junction and tandem flexible devices were achieved, comparable with those of the ITO‐based rigid counterparts. Also, flexible OSCs exhibited outstanding mechanical stability with high retention of 95% of the initial PCEs after 1000 times bending (radius = 5 mm). Note that very recently, the OPV area has witnessed a great step forward in the last few years mainly due to the new emerging star acceptor Y6 and its derivatives. Thus, it is natural and important to study the performance of flexible OPV devices with these outstanding new materials. Using these FlexAgNEs, the flexible OPV devices with PM6:BTP‐4F‐12 blends obtained a high PCE of 15.6%, proving that FlexAgNEs in efficient systems still have a good universality.^[^ ^124^ ^]^ In addition, the flexible devices displayed superior and robust mechanical stability, and even with >83% PCEs retention at bending radius close to 0 mm in both bending directions. Recently, Li's group proposed a welding strategy to design FTEs, consisting of the embedded AgNWs plastic substrate and AgNWs:Al‐doped ZnO (AZO) upper layer (Figure 6e).^[^ ^27^ ^]^ Due to the capillary force and secondary growth of the AZO, the FTEs got smooth surface and reduced junction‐site resistance (18 Ω sq^−1^ with transmittance of ≈95%). Using PM6:Y6 blend, flexible OSCs obtained a PCE of 15.21%. The flexible OSCs retained 93.7% of the initial efficiency after 1200 cycles outward bending (radius = 4 mm).

In addition, due to the great success of AgNWs‐based FTEs in flexible OSCs, some efforts have been made in further improving device performance and fabricating large‐area and ultraflexible devices in recent years. Tang and coworkers reported a biomimetic FTE, which is composed of ZnO protecting layer, conductive AgNWs, light‐scattering polystyrene spheres, and PI substrate.^[^ ^62^ ^]^ The biomimetic FTE offers high transmittance (88.2%), low resistance (23.4 Ω sq^−1^), and small surface roughness (2.4 nm). The inverted flexible OSCs with PM6:N3:PC_71_BM blend achieved a PCE of 16.1%, and kept 13.7% after 5000 cycles bending with a small radius (1.0 mm). Moreover, Ma et al. presented a gravure printing technology to fabricate large‐area FTEs via semiembedding AgNWs into the low‐temperature processed colorless polyimide (cPI) (Figure 6f).^[^ ^60^ ^]^ The prepared FTEs exhibited resistance of 10–30 Ω sq^−1^ with the transmittance of 88–91%. Using PM6:Y6 blend, PCEs of 15.28% and 13.61% for small‐area and large‐scale flexible OSCs (0.04 and 1 cm^2^) were achieved, respectively. Compared with spin coating, the gravure printing exhibited improved uniformity, which could be ascribed to better ink distribution on the substrate. These results proved that it was an ideal method to prepare large‐area flexible OSCs via a gravure printing process. Furthermore, Zhou and coworkers designed a novel interlayer of Zn^2+^‐chelated polyethylenimine (PEI‐Zn) to fabricate ultraflexible OSCs with a 1.3 μm‐thick PEN/AgNWs electrode.^[^ ^59^ ^]^ The PEI‐Zn offered a mechanical bending strain >2 times higher than that of ZnO, and had good chemical compatibility with NFA materials. Finally, with PM6:Y6 active layer, the inverted ultraflexible OSCs achieved a record PCE of 15.0%. In addition, the ultraflexible OSCs exhibited almost unchanged PCE after 100 cycles compression‐flat deformation.

Therefore, AgNWs‐based FTEs have demonstrated better optoelectrical properties and mechanical flexibility in comparison with FTEs based on ITO, graphene, CNTs, and PEDOT:PSS. Nevertheless, there are still some issues that need to be solved, e.g., AgNWs migration and oxidation and so on. It may be useful to semiembed AgNWs in a thin polymer layer to effectively suppress the migration and oxidation of AgNWs and achieve high‐quality AgNWs FTEs.^[^ ^125^ ^]^

## 6 Flexible OSCs Based on Ultrathin Metal Films and Metal Meshes

Ultrathin metal films and metal mesh electrodes have been considered as promising candidates for FTEs owing to their combined good features of high conductivity of metals and excellent mechanical flexibility of ultrathin films (<10 nm).

### 6.1 Ultrathin Metal Films

Ultrathin metal films have attracted increasing attention as FTEs, which combined characteristics of good transparency, low sheet resistance, and superior flexibility due to the excellent ductility of metal. As early as 2008, Guo's group reported transparent ultrathin metal electrodes in rigid OSCs via a nanoimprinted process. This electrode showed high conductivity, and the relative devices exhibited similar PCE to ITO‐based counterparts.

**Figure 7:**
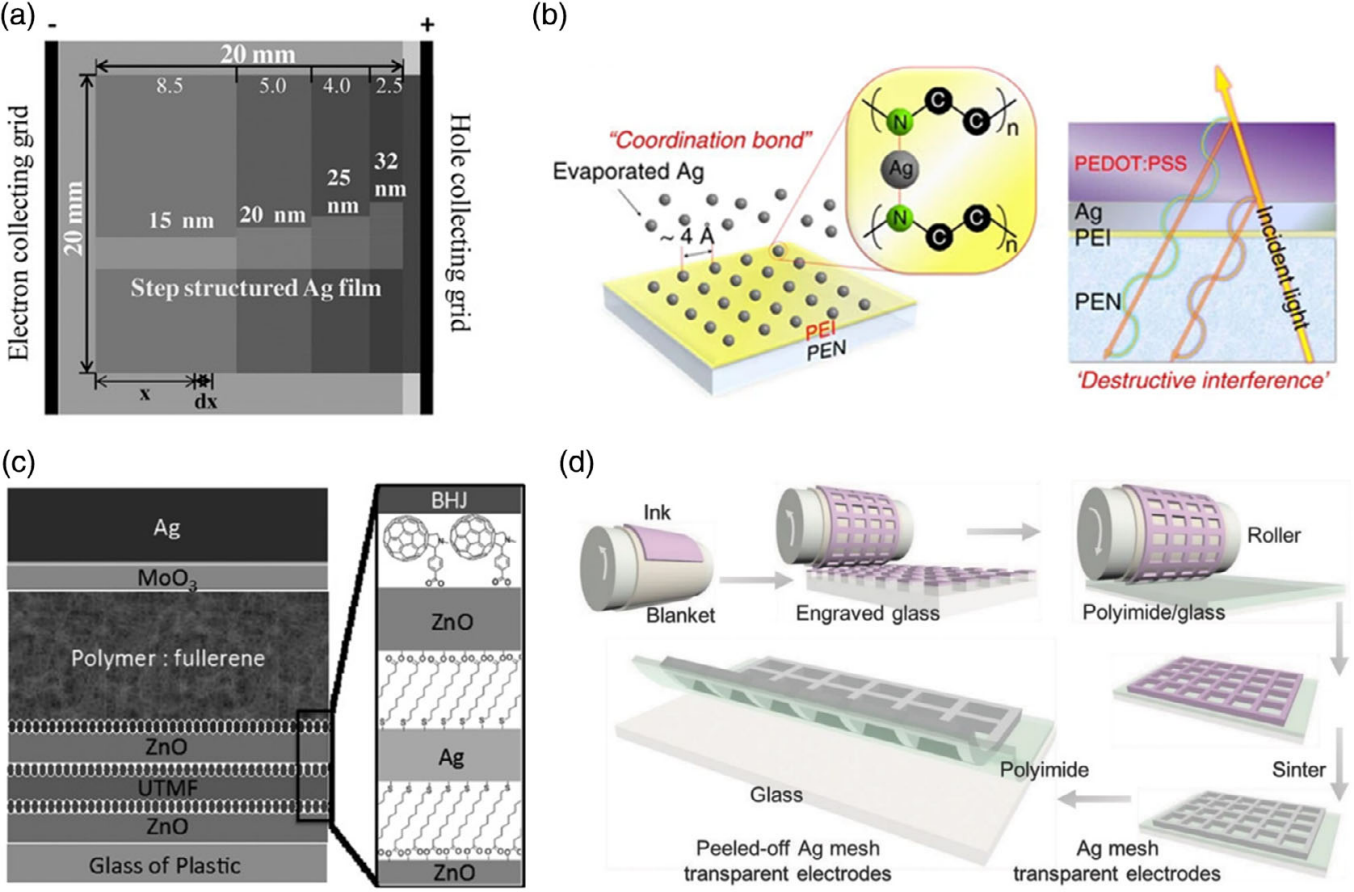
a) Top‐view of the device structures for large‐area solar cells (2 cm × 2 cm) with the thickness step‐structured Ag layer of 15, 20, 25, and 32 nm thickness from left to right. Reproduced with permission.^[^ ^127^ ^]^ Copyright 2015, Wiley‐VCH. b) Conceptual diagram for the growth mechanism of the Ag film with the PEI nucleation inducer, and the destructive interference in the Ag film with the PEDOT:PSS antireflective layer. Reproduced under the terms of the CC‐BY 4.0 license.^[^ ^28^ ^]^ Copyright 2015, The Authors, published by Springer Nature. c) Schematic drawing of the PSC devices and the molecular structure of MUA and C60‐SAM used for interfacial modifications. Reproduced with permission.^[^ ^126^ ^]^ Copyright 2014, Wiley‐VCH. d) Schematic for the fabrication process of ultrathin Ag mesh transparent electrodes. Reproduced with permission.^[^ ^65^ ^]^ Copyright 2018, Wiley‐VCH.

However, it is challenging to realize high‐quality ultrathin metal films due to Volmer–Weber‐growing mode of the metal film, delivering a number of island‐like grains.^[^ ^126^ ^]^ Thus, it is crucial to carefully modify the evaporation process. In 2015, Chen et al. designed a conductance‐ and thickness‐gradient ultrathin Ag film as FTEs (see **Figure** **7**a).^[^ ^127^ ^]^ The reduced energy loss of the FTEs could be attributed to the balanced sheet resistance and light trapping. With these, utilizing PTB7‐Th:PC_71_BM blend, a PCE of 7.15% was achieved for large‐area (4 cm^2^) flexible OSCs, sustaining 80% of small‐area (0.052 cm^2^) devices. In addition, an efficient strategy to assist the ultrathin metal film growth and restrain metal atom diffusion has been reported, which is preevaporating seeds. The wetting behavior of the pretreated substrates could be finely tuned, resulting from the increased chemical interactions of metal atoms with seeds. In 2015, Lee's group reported a polymer–metal hybrid electrodes via preintroducing a solution‐processed PEI with functional amine groups on plastic PEN substrates (Figure 7b)^[^ ^28^ ^]^. The PEI layer offers ideal metal‐nucleation sites, leading to a high‐standard FTE with outstanding performances (resistance <10 Ω sq^−1^ with transmittance >95%). These features were attributed to the surface modification of plastic substrates with a nonconjugated amine‐containing polyelectrolyte, which provided uniform metal nucleation sites. Using PTB7‐Th:PC_71_BM blend, flexible OSCs achieved a high PCE of 9.9%, similar to rigid ITO‐based counterparts (10.1%). Recently, Bi and coworkers designed a composite FTE of S‐1805/Ag/Au.^[^ ^128^ ^]^ The island‐like grains of Au film (4.4 nm) were effectively suppressed with good wettability of thin Ag layer (0.6 nm), and also the chemical bonding effect of S‐1805. This electrode exhibited low surface roughness, low resistance (78 Ω sq^−1^) and relatively high transmittance (78%). With PCDTBT:PC_71_BM blend, the flexible OSCs got a PCE of 5.21%, similar to ITO‐based rigid counterparts (5.70%). Furthermore, the PCE slightly decreased after >2000 cycles bending with a radius of ≈7.5 mm.

To improve the ultrathin metal films performance, oxide–metal–oxide or dielectric–metal–dielectric structure electrodes have been presented in ultrathin metal electrodes. An ultrathin metal film with high reflective index is inserted in two highly refractive oxide layers. It can effectively improve the transmittance of FTEs and also prevent the oxidization of metal with the oxygen and water vapor.^[^ ^129^ ^]^ In 2014, Jen and coworkers created ZnO/Ag/ZnO electrodes, which were modified with the self‐assembled monolayer (Figure 7c).^[^ ^126^ ^]^ This PEN/ZnO/Ag/ZnO electrode showed smooth surface, excellent conductivity (8.61 Ω sq^−1^), and good transmittance (80%). With PIDT‐PhanQ:PC_71_BM blend, the flexible OSCs yielded a PCE of 6.04%, comparable with ITO‐based rigid counterparts (6.38%). Moreover, a high retention (92%) of the initial efficiency was achieved after 200 times bending (radius = 0.55 mm), indicating excellent bending performance of the ZnO/Ag/ZnO electrodes. Later, Yun's group prepared ZnO/AgO_*x*_/ZnO (O/Ag = 3.4 wt%) via oxygen doping of Ag.^[^ ^130^ ^]^ The AgO_*x*_ layer showed smaller optical absorbance and reflections, lower sheet resistance, and more continuous and smooth morphology in comparison with pure Ag layer with the same thickness. The ZnO/AgO_*x*_/ZnO electrodes achieved resistance of 20 Ω sq^−1^ with transmittance of 91%. Based on PTB7‐F20:PC_71_BM blend, the flexible OSCs achieved a PCE of 6.34%, higher than that of the devices based on ZnO/Ag/ZnO (5.65%). Moreover, the PCE of the flexible OSCs almost unchanged until bending radius decreased to 1.1 mm. In addition, other electrodes with OMO or DMD structures that offer good optoelectrical performance and are adaptable in flexible OSCs have been demonstrated, such as WO_3_/Ag/WO_3_,^[^ ^131^ ^]^ MoO_3_/LiF/MoO_3_/Ag/MoO_3_,^[^ ^132^ ^]^ TiO_2_/Ag/ITO,^[^ ^133^ ^]^ SnO_*x*_/Ag/SnO_*x*_,^[^ ^134^ ^]^ and MoO_*x*_/Ag/ZnS.^[^ ^135^ ^]^

Moreover, Zhou et al. created and reported a design of bottom metal electrode and top transparent electrode to realize high‐performance large‐area (10.5 cm^2^) flexible tandem OSCs.^[^ ^136^ ^]^ Compared with the corresponding single‐junction devices, the flexible tandem OSCs show higher PCEs and improved manufacturing yield. The large‐area (10.5 cm^2^) flexible tandem device maintained 82% of the small‐area counterparts. Using the series architecture method is more likely to reduce the undesired shunt resistance effect. The increased number of sequential depositions of various organic layers in the tandem devices, provided that the inherent defects in each layer are not aligned in the vertical direction, will increase the total shunt resistance of the solar cell, thereby improving yield and performance.

### 6.2 Metal Meshes

Although the ultrathin metal films have been extensively used in flexible OSCs, as the thickness of ultrathin metal film decreases, its resistance will increase sharply, it is still hard to achieve high‐performance ultrathin metal electrodes. To address this issue, metal meshes or patterned metal grids have been presented as promising candidates to ITO. These electrodes can be fabricated with diverse techniques, such as thermal evaporation, nanoimprint lithography,^[^ ^137^ ^]^ gravure offset and inkjet printing,^[^ ^138^, ^139^ ^]^ and dry etching.^[^ ^140^ ^]^ Via tuning the mesh or grid parameters, including line width, spacing, aspect ratio, and depth, the optoelectrical properties can be optimized.^[^ ^141^ ^]^

In 2007, Inganäs's group first fabricated a FTE based on Ag grids via a soft lithographic metal deposition process, forming line width and spacing of 40 and 600 μm, respectively.^[^ ^142^ ^]^ After coating of glycol‐PEDOT:PSS layer on the Ag grids, the roughness dramatically reduced, and also the resistance decreased from 350 to 70 Ω sq^−1^. Based on the glycol‐PEDOT:PSS/Ag grids electrodes, the flexible OSCs with APFO‐Green‐5:PC_61_BM blend got a PCE of 1.0%. In 2013, Li et al. designed high‐resolution hexagonal Ag grid electrodes embedded in PET substrate with the line width of 3 μm, which offered transmittance of 85% and very low resistance of 0.5 Ω sq^−1^.^[^ ^143^, ^144^ ^]^ Later, a composite flexible PET/Ag grid/PH1000 electrode was prepared with low resistance of 1.2 Ω sq^−1^ with transmittance of 80%. Large‐area (1.21 cm^2^) flexible OSCs using PTB7:PC_71_BM blend exhibited a PCE of 5.85%. Recently, they further reported a composite electrode, composed of Ag mesh, PH1000, and AgNWs (the PH1000 doping with 20 wt% AgNWs).^[^ ^64^ ^]^ This electrode simultaneously offered low resistance (6 Ω sq^−1^), high transmittance (86%), and smooth surface. With Ag mesh/PH1000:AgNWs electrodes, flexible OSCs using PM6:IT‐4F blend showed a PCE of 12.07%, similar to the rigid ITO‐based counterparts (12.94%). Moreover, Lee’ group fabricated a junction‐free silver nanonetworks (AgNNs) electrode via a dry etching using nanoscale shadow masks.^[^ ^140^ ^]^ For an ultrathin Ag layer, the controllable density of AgNNs can bring about low sheet resistance and high transparency. Finally, the patterned AgNNs electrodes offered a high transmittance of 94.4% with resistance as low as 2.4 Ω sq^−1^. Flexible OSCs based on PTB7‐Th:IEICO‐4F blend showed a PCE of 10.6%, and offered a high retention (94.3%) of initial efficiency after 3000 cycles bending (strain: 3.13%). In addition, Someya and coworkers fabricated Ag mesh electrodes with uniform and ultrathin thickness of 100 nm via a reverse‐offset printing method (Figure 7d).^[^ ^65^ ^]^ A low resistance of 17 Ω sq^−1^ with high transmittance of 93.2% was simultaneously obtained, which was superior to those of the sputtered ITO electrodes. This electrode also exhibited superior mechanical durability, e.g., only 16.6% and 10.6% increases in sheet resistances under 50% compression and 500 cycles stretch/release strain, respectively. Based on the Ag mesh electrodes, the flexible OSCs with PTzNTz:PC_71_BM blend got a PCE of 8.3%, and offered a high yielding (>90%).

Thus, the ultrathin metal films and metal meshes have been demonstrated in flexible OSCs because of their low resistance and superior mechanical durability. Nevertheless, there still remain some challenges. For ultrathin metal films, it is essential to control the metal growth and suppress Volmer–Weber growth. For example, the substrate can be chemically modified to avoid the island‐like metal grains. Moreover, emerging highly refractive oxide layers should be explored to prevent the metal oxidation and corrosion. For metal mesh, developing hybrid metal mesh electrodes may be an effective strategy to achieve high‐quality FTEs with superior optoelectrical properties and excellent mechanical stability.

## 7 Conclusions and Outlook

In summary, flexible OSCs are sorted out and further thoroughly discussed from the perspective of the related FTEs (including ITO, carbon nanomaterials, conducting polymers, metal nanowires, ultrathin metal films, and metal meshes) used in devices. Meanwhile, the detailed discussion on different FTEs, photovoltaic materials, and device performance in flexible OSCs uncovers the underlying structure–property relationship. Considering that there still remains big room for improvement for flexible OSCs, it is of great necessity for synergistic efforts in optimization of FTEs, photoactive materials, and electrode buffer layers. With this in mind, the following directions and endeavors are given for future flexible OSCs development:

1) High‐quality FTEs. FTEs should possess the combined good features simultaneously (including minimum resistance, high transparency, smooth surface, good stability, superior flexibility, and facile but effective approach), rather to have the outstanding performance only in some aspects but others very limited. 2) Efficient photoactive materials. Exploring highly efficient new photoactive materials is essential to further improve device performance. For example, it may be an effective strategy to design new NFA materials and matching donor materials via rational design strategies (including central units, side chains, and terminal groups). 3) Photoactive layers with excellent mechanical durability. For flexible OSCs, it is of great necessity and importance to design photoactive layer materials with good tensile modulus and elongation at break. It may be useful to develop high‐performance all‐polymer photoactive layers to provide excellent mechanical and operating stability.^[^ ^80^, ^145^, ^146^ ^]^ 4) Low‐temperature solution‐processed electrode buffer layers. To be compatible with the roll‐to‐roll printing technology and avoid damage the plastic substrates, solution‐processed low‐temperature interface materials should be developed. Moreover, exploring electrode buffer layers with low trap states and high conductivity is beneficial to realize thick‐film interface layers, which is an important factor for mass production. 5) Easy large‐scale printing techniques. To adapt to industrial production, developing facile but effective large‐scale printing methods is essential.

## Conflict of Interest

The authors declare no conflict of interest.
